# Immunomodulatory activity of heat‐killed *Lacticaseibacillus paracasei* MCC1849 based on the activation of plasmacytoid dendritic cells in the peripheral blood of healthy adults

**DOI:** 10.1002/fsn3.4009

**Published:** 2024-02-07

**Authors:** Yiran Li, Takahiro Aoki, Sadahiro Iwabuchi, Satoshi Arai, Noriyuki Iwabuchi, Hideki Motobayashi, Miyuki Tanaka, Shinichi Hashimoto

**Affiliations:** ^1^ Innovative Research Institute, R&D Division, Morinaga Milk Industry Co., Ltd. Zama Kanagawa Japan; ^2^ Department of Molecular Pathophysiology Wakayama Medical University Wakayama Wakayama Japan; ^3^ Second Department of Surgery Wakayama Medical University Wakayama Wakayama Japan

**Keywords:** immunomodulatory, *Lacticaseibacillus paracasei*, plasmacytoid dendritic cells, postbiotics

## Abstract

Probiotics are widely used in food for their health benefits to the host. Inactivated probiotics also reportedly improve the intestinal environment and immune regulation. Our previous studies showed that heat‐killed *Lacticaseibacillus paracasei* MCC1849 (hk‐MCC1849) effectively induced IL‐12 production in mouse spleen cells and significantly reduced cold symptoms in clinical trial subjects. To further elucidate the mechanism of host immune regulation by hk‐MCC1849, human peripheral blood mononuclear cells (PBMCs) were cocultured with hk‐MCC1849. The Toll‐like receptor 9 ligands CpG‐ODN 2216 and hk‐MCC1849 and the heat‐killed *Lacticaseibacillus rhamnosus* ATCC53103 were used as positive and negative controls, respectively. The results showed that, compared with the control, hk‐MCC1849 significantly increased the expression of the plasmacytoid dendritic cell (pDC) marker CD86 (*p* < .0001) and the pDC marker HLA‐DR (*p* < .001) in PBMCs. The expression levels of the *IL‐12p40*, *IFNα*, *IFNα1*, *IFNγ*, and *ISG15* genes were significantly increased after coculture with hk‐MCC1849 (*p* < .05, *p* < .05, *p* < .05, *p* < .05, and *p* < .05, respectively, vs. control). Furthermore, to confirm whether hk‐MCC1849 directly interacted with pDCs, DCs were enriched with PBMCs following 24 h of coculture with hk‐MCC1849. Phagocytosis of fluorescently labeled hk‐MCC1849 by pDCs was observed, and there were significant increases in CD86 (*p* < .05) and HLA‐DR (*p* < .0001) expression in pDCs. These results suggest that hk‐MCC1849 exerts a potential immunomodulatory effect on the host through the activation of peripheral pDCs.

## Graphical abstract

Heat‐killed *Lacticaseibacillus paracasei* MCC1849 was phagocytosed by pDCs after 24‐h coculture and significantly upregulated the expression of surface molecules, CD86 and HLA‐DR, on pDCs derived from human peripheral blood. These results suggest that hk‐MCC1849 exerts a potential immunomodulatory effect on the host through the activation of peripheral pDCs.

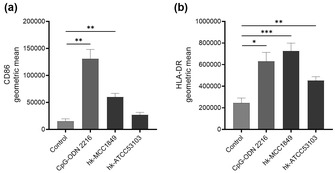

## 1 INTRODUCTION

Probiotics are live microorganisms that live in the gastrointestinal tract and have health benefits for the host/consumer upon the administration of sufficient amounts (Hill et al., 2014). The administration of postbiotics, such as heat‐killed bacteria, following probiotic treatment, has emerged as a new area of study. Sashihara et al. (2006) reported that heat‐killed *Lactobacillus* species, as well as live probiotics, could alleviate allergic disease. Many studies have demonstrated that inactivated lactic acid bacteria have various effects, including modification of the host microbiota and immune responses (Salminen et al., 2021).

*Lacticaseibacillus paracasei* MCC1849 was isolated from the adult gastrointestinal tract, and heat‐killed MCC1849 (hk‐MCC1849) has been demonstrated to lower the risk of contracting the common cold in susceptible adults and modulating immune responses to the influenza vaccine in elderly individuals without side effects (Maruyama et al., 2016; Murata et al., 2018). In our previous study, oral administration of hk‐MCC1849 to mice increased the intestinal IgA concentration and the gene expression levels of *Il‐10*, *Il‐12*, and *Il‐21* in Peyer's patches (PPs) via T‐follicular helper cell induction (Arai et al., 2018). This result suggested that the administration of hk‐MCC1849 could regulate the host immune response through PPs; however, the target cell with which hk‐MCC1849 interacts remains still unknown.

PPs are important mucosal lymphoid organs in the intestine that undergo transcytosis mediated by M cells for antigen recognition (Mabbott et al., 2013). Plasmacytoid dendritic cells (pDCs) play an important role in capturing antigens from the lumen and activating humoral and/or cell‐mediated immune reactions (Crother et al., 2012). By expressing the Toll‐like receptors (TLRs) TLR7 and TLR9, pDCs can rapidly respond to viral or nonviral pathogens by producing large amounts of type I interferon (IFN) through the MyD88/IRF7 signaling pathway (Cella et al., 2000). The most highly expressed human type I IFNs are IFN‐α, which has 13 subtypes, and one subtype of IFNβ, followed by trace amounts of IFNε, IFNκ, and IFNω (Ali et al., 2019). Among human peripheral blood mononuclear cells (PBMCs), pDCs are major type I IFN‐producing cells that contribute more than 95% of IFN production upon viral challenge (Siegal et al., 1999). After acute viral infection, type I IFNs can accelerate the maturation of DCs into antigen‐presenting cells, activate B cells and T cells, and promote their differentiation (Crouse et al., 2015; Kiefer et al., 2012; McNab et al., 2015). IFNα and IFNβ are essential for the stimulation of innate and acquired immune responses against viral invasion (Theofilopoulos et al., 2005).

Although previous animal tests and clinical studies have demonstrated that hk‐MCC1849 can improve acquired immune responses in mice and immunomodulatory effects in humans, the interaction between hk‐MCC1849 and human‐derived immune cells is still not well understood. In this study, we used PBMCs derived from the peripheral blood of healthy adults to investigate whether hk‐MCC1849 can enhance host immunity by evaluating the activation of pDCs and the expression levels of cytokines.

## 2 MATERIALS AND METHODS

This study was conducted in accordance with the current revision of the Declaration of Helsinki (2013) and the ethical guidelines for medical and health research involving human subjects (2015). The research protocol and informed consent form were approved by the Institutional Review Board (IRB) of Wakayama Medical University, Japan (Approval No. 3345). Written informed consent was obtained from healthy adult donors.

### 2.1 Preparation of PBMCs

Peripheral blood was collected from each participant in Vacutainer test tubes containing heparin sodium (Terumo, Japan). Peripheral blood was diluted with an equal volume of 0.9% NaCl. Eighteen milliliters of diluted blood was carefully layered over 10 mL of Lymphoprep in a Lymphoprep™ Tube (Serumwerk, Germany), and the tube was centrifuged at 800× *g* for 20 min at 25°C. After centrifugation, the mononuclear cells were collected from a distinct band at the sample/medium interface as described in the separation procedure. The harvested mononuclear cell fraction was diluted four‐fold with 0.9% NaCl and centrifuged at 400× *g* for 5 min at 25°C. The supernatant was removed, and the red blood cells were hemolyzed using ammonium chloride solution (eBioscience, USA) for 10 min at 25°C. After hemolysis, the cells were washed twice with cold D‐PBS (Fujifilm, Japan), counted, and used as PBMCs for the following experiments.

### 2.2 Preparation of microorganisms

*Lacticaseibacillus paracasei* MCC1849 (strain LAC‐Shield) and *Lacticaseibacillus rhamnosus* ATCC53103 were obtained from stock cultures maintained at the Morinaga Culture Collection (MCC; Morinaga Milk Industry Co., Ltd., Japan) and purchased from the American Type Culture Collection (ATCC; Manassas, VA, USA), respectively. These organisms were cultured for 16 h at 37°C in Lactobacilli‐MRS broth (DIFCO, Mich., USA). Bacteria were collected via centrifugation at 4000× *g* and washed with sterile distilled water three times to completely remove the medium. Bacteria were heat inactivated at 95°C for 30 min, after which the number of bacteria was determined via microscopic counting via a bacteria counter. Heat‐killed bacteria were suspended in RPMI‐1640 medium and adjusted to an appropriate concentration. No visible colonies were observed after 48 h of culture at 37°C using MRS agar medium.

### 2.3 Coculture of PBMCs and heat‐killed bacteria

PBMCs were suspended in RPMI‐1640 supplemented with 25 mM HEPES, 10% fetal bovine serum, 100 U/mL penicillin, and 100 μg/mL streptomycin. PBMCs (5 × 10^5^ cells/well) were incubated in a 24‐well plate (Corning, USA) and cocultured with 1 × 10^6^ cells/mL hk‐MCC1849 or heat‐killed *Lacticaseibacillus rhamnosus* ATCC53103 (hk‐ATCC53103) and Cpg‐ODN 2216 (1 μM) (InvivoGen, USA). A no‐addition well was used as the control. PBMCs were cocultivated at 37°C under 5% CO2 in humid conditions.

### 2.4 DC enrichment and coculture

The PBMCs prepared in Section 2.1 were resuspended in RoboSep Buffer (STEMCELL, USA) and used immediately. DCs were isolated by negative immunoselection with an EasySep Human Pan‐DC Pre‐Enrichment Kit (STEMCELL, Canada) according to the manufacturer's instructions. To increase the DC ratio, the magnetic particle binding step was performed twice. After isolation, the cells were resuspended in RPMI‐1640 supplemented with 25 mM HEPES, 10% fetal bovine serum, 100 U/mL penicillin, and 100 μg/mL streptomycin. The cells (1 × 10^5^ cells/well) were incubated in a 384‐well plate (Corning, USA) and cocultured with 1 × 10^6^ cells/well of hk‐MCC1849 or hk‐ATCC53103 at 37°C under 5% CO2 and humid conditions. A no‐addition well was used as the control.

### 2.5 FACS analysis

After 24 h of coculture, the PBMCs or DCs were washed with cold D‐PBS and stained to identify dead cells using a Horizon Fixable Viability Stain 780 (BD, USA). The cells were washed with staining buffer (BD, USA) twice and treated with Human BD Fc Block (BD, USA) on ice to prevent the false‐positive binding of antibodies. Afterward, the antibodies were conjugated to the cells by staining them with FITC‐labeled anti‐human CD304 (clone no. U21‐1283), PE‐Cy7‐labeled anti‐human CD123 (clone no. 7G3), APC‐labeled anti‐human CD86 (clone no. 2331), and PE‐labeled anti‐human HLA‐DR (clone no. G46‐6) (BD, USA) for 20 min on ice in the dark. For DC ratio measurements, cells were stained with FITC‐labeled anti‐human CD4 (clone no. OKT4), CD8 (clone no. RPA‐T8), CD14 (clone no. M5E2), CD16 (clone no. 3G8), CD19 (clone no. HIB19), CD20 (clone no. 2H7), CD56 (clone no. HCD56), PE‐Cy7‐labeled anti‐human CD123 (clone no. S18016F), APC‐labeled anti‐human HLA‐DR (clone no. L243), and PE‐labeled anti‐human CD11c (clone no. Bu15) (BioLegend, USA). For bacterial phagocytosis analysis, DCs were stained with PE‐Cy7‐labeled anti‐human CD123 (clone no. 7G3) and APC‐labeled anti‐human CD304 (clone no. Neuropilin‐1). The stained cells were washed twice with staining buffer and fixed with cytofix buffer (BD, USA) for FACS analysis. The data were processed and obtained using FlowJo ver. 7.6 (BD, USA).

### 2.6 RNA extraction and real‐time quantitative reverse transcription PCR (qRT‐PCR) analysis

Total RNA was extracted from the cells after 4 h of coculture using NucleoSpin® RNA Plus (Takara, Japan). Complementary DNA was prepared using PrimeScript RT Master Mix (Takara, Japan) according to the manufacturer's protocol. qRT–PCR was performed using SYBR Premix Ex Taq (Takara, Japan) in a 7500 Fast Real‐time PCR System (Applied Biosystems, USA). The amplification program was as follows: initial hold at 95°C for 30 s, followed by 40 cycles of 95°C for 3 s and 60°C for 30 s. The sequences of the primers that were designed and synthesized are listed in Table 1. The 2^−ΔΔCt^ method was applied to calculate the relative quantity of gene expression normalized to that of the housekeeping gene β‐actin.

**TABLE 1:** Primers used in this study.

Gene	Forward primer (5′‐3′)	Reverse primer (5′‐3′)
*β‐Actin*	GAGCGGGAAATCGTGCGTGACATT	TGCCCAGGAAGGAAGGCTGGAAGA
*IL12p40*	CCTGCTGGTGGCTGACGACAAT	CTTCAGCTGCAAGTTGTTGGGT
*IFNα*	GACCAGGAGACACGGAATGT	GATGTAATCCTTGCCGTCGT
*IFNα1*	GCAAGCCCAGAAGTATCTGC	ACTGGTTGCCATCAAACTCC
*IFNα2*	AAATACAGCCCTTGTGCCTGG	GGTGAGCTGGCATACGAATCA
*IFNβ*	AAGGCCAAGGAGTACAGTC	ATCTTCAGTTTCGGAGGTAA
*IFNγ*	TGACCAGAGCATCCAAAAGA	CTCTTCGACCTCGAAACAGC
*ISG15*	GCGGGCAACGAATTCCAGGTGT	TCGCATTTGTCCACCACCAGCA

### 2.7 Analysis of bacterial phagocytosis

The hk‐MCC1849 prepared in Section 2.2 was resuspended in 100‐mM carbonate buffer (pH 9.6). FITC isomer I (Sigma, USA) was then added to the cells at a concentration of 0.1 mg/mL, and the mixture was incubated at room temperature for 60 min in the dark. The hk‐MCC1849 cells were washed with sterile PBS and resuspended in RPMI‐1640 medium. FITC‐labeled hk‐MCC1849 was added to 384‐well plates and incubated with DCs for 24 h at 37°C under 5% CO2. For FACS analysis, cells were stained as described in Section 2.5. The cells were washed twice with PBS, and the nuclei were stained with 2 μg/mL 4′,6‐diamidino‐2‐phenylindole (Dojindo, Japan) at 37°C for 15 min in the dark. Images were acquired using a laser scanning confocal fluorescence microscope (Olympus Fluoview FV3000, Japan), and detailed 3D images were constructed using Olympus FV31S‐SW software ver. 2.5.1 (Olympus, Japan).

### 2.8 Statistical analysis

All the data are presented as the mean and standard error. A repeated measures ANOVA was used to compare the cell‐surface marker activities and relative gene expression levels between groups, and the results were analyzed with SPSS version 26 (IBM Corp., USA). To assess the statistical significance of the differences in gene expression among the experimental groups, ΔCt values were calculated according to the standard propagation of error methods described by Livak and Schmittgen (2001).

## 3 RESULTS

### 3.1 Donor characteristics

Peripheral blood was initially collected from 10 healthy adult donors for PBMC isolation and DC enrichment. The average age of the 10 donors was 39.4 ± 10.8 years (mean ± standard deviation; SD), and the age range was 23–59 years. Subsequently, two samples were excluded due to the aggregation of PBMCs, which may have been caused by severe allergic symptoms. The average age of the remaining eight donors was 38.9 ± 11.3 years (mean ± SD), and the age range was 23–59 years. The sex ratio of the donors was 5:3 (male: female).

### 3.2 Hk‐MCC1849 activated the surface markers of pDCs in PBMCs

**FIGURE 1:**
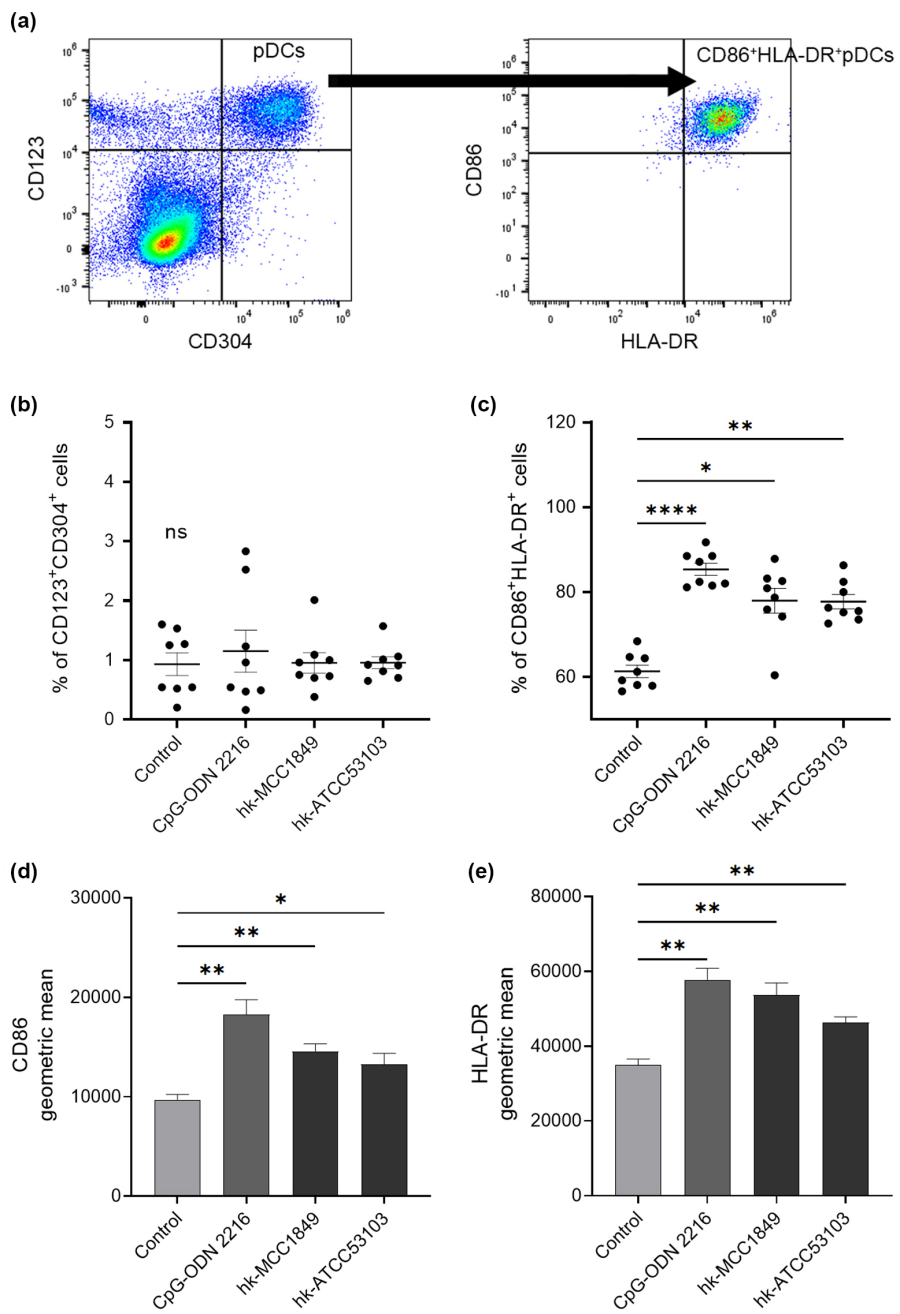
Effects of Cpg‐ODN 2216, hk‐MCC1849, and hk‐ATCC53103 on the activation of pDC markers. Gating strategy in flow cytometry to observe pDCs in PBMCs (a). The percentages of CD123^+^CD304^+^ cells in total live cells (b) and the percentages of CD86^+^HLA‐DR^+^ cells in pDCs (c) were evaluated by FlowJo. The geometric means of CD86 (d) and HLA‐DR (e) activation in total pDCs. All the data are expressed as the mean ± SE (*n* = 8). *p < .05, **p < .01, and ****p < .0001 indicate a significant difference versus the control. ns indicates no significant difference observed among the treatments.

Flow cytometry was used for cell‐surface marker analysis, and the gating strategy is shown in Figure 1a. First, single mononuclear cells were selected. Then, the live cells were separated, and the live cell ratio was confirmed to indicate that the addition of Cpg‐ODN 2216 or heat‐killed bacteria would not affect the viability of mononuclear cells. Subsequently, CD123^+^CD304^+^ cells were defined as pDCs. CD86^+^HLA‐DR^+^ pDCs were gated to assess the activation of surface markers. The percentage of total live CD123^+^CD304^+^ cells was calculated, and there was no difference among the treatment groups (Figure 1b). Although coculture with heat‐killed lactic acid bacteria or Cpg‐ODN 2216 did not affect the ratio of pDCs in PBMCs, a significant increase in the CD86^+^HLA‐DR^+^ ratio among all pDCs was observed. Cpg is a potent TLR9 ligand that strongly initiates the innate immune response and induces an adaptive immune response. In the present study, Cpg‐ODN 2216 significantly induced the expression of the surface markers CD86 (*p* < .01) and HLA‐DR (*p* < .01) (Figure 1d,e). Heat‐killed lactic acid bacteria were added to the medium and cocultured with PBMCs for 24 h. The addition of hk‐MCC1849 and hk‐ATCC53103 significantly increased the ratio of CD86^+^HLA‐DR^+^ cells to total pDCs (*p* < .05 and *p* < .01, respectively) (Figure 1c), and the expression levels of the surface markers CD86 (*p* < .01 and *p* < .05, respectively) and HLA‐DR (*p* < .01 and *p* < .01, respectively) on pDCs were significantly elevated compared with those on the control (Figure 1d,e).

### 3.3 Heat‐killed *Lacticaseibacillus paracasei* MCC1849 promoted type I interferon gene expression

**FIGURE 2:**
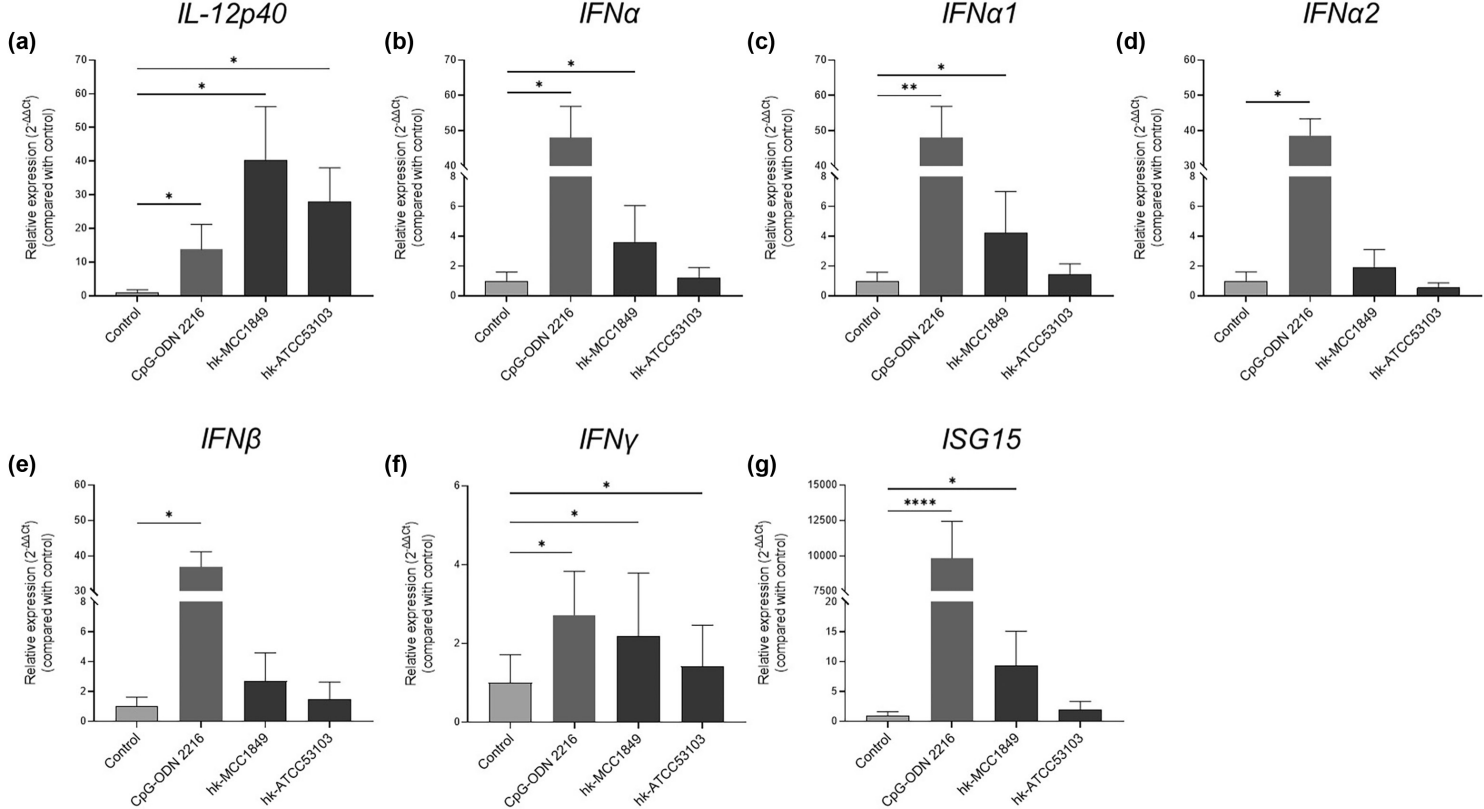
Effects of Cpg‐ODN 2216, hk‐MCC1849, and hk‐ATCC53103 on the expression levels of *IL‐12p40* (a), *IFNα* (b), *IFNα1* (c), *IFNα2* (d), *IFNβ* (e), *IFNγ* (f), and *ISG15* (g) in human PBMCs by qRT‐PCR analysis. All the data are expressed as the mean ± SE (*n* = 8). *p < .05, **p < .01, and ****p < .0001 indicate a significant difference versus the control.

To further determine whether heat‐killed MCC1849 affects the immune response of PBMCs, the relative gene expression levels of *IL‐12p40* and type I IFNs in PBMCs were analyzed via qRT‐PCR. Specifically, we examined the expression of the *IL‐12p40*, *IFNα*, *IFNα1*, *IFNα2*, *IFNβ*, *IFNγ*, and *ISG15* genes after coculture with Cpg‐ODN 2216 and heat‐killed lactic acid bacteria for 4 h. Cpg‐ODN 2216 strongly induced the expression of the type I IFN genes and *ISG15* compared with the control (Figure 2). After stimulation with heat‐killed lactic acid bacteria, both hk‐MCC1849 and hk‐ATCC53103 increased the expression levels of *IL‐12p40* (*p* < .05 and *p* < .05, respectively) (Figure 2a). Coculture with hk‐MCC1849 significantly increased the relative expression levels of *IFNα* (*p* < .05), *IFNα1* (*p* < .05), *IFNγ* (*p* < .05), and *ISG15* (*p* < .05) compared with those in the control group (Figure 2b,c,f,g). No significant changes in the expression levels of *IFNα2* or *IFNβ* were observed (Figure 2d,e). Although coculture with hk‐ATCC53103 significantly upregulated the expression of *IFNγ* (*p* < .05) compared with that in the control group (Figure 2f), no other changes in type I IFN expression were found.

### 3.4 Phagocytosis of hk‐MCC1849 by pDCs

**FIGURE 3:**
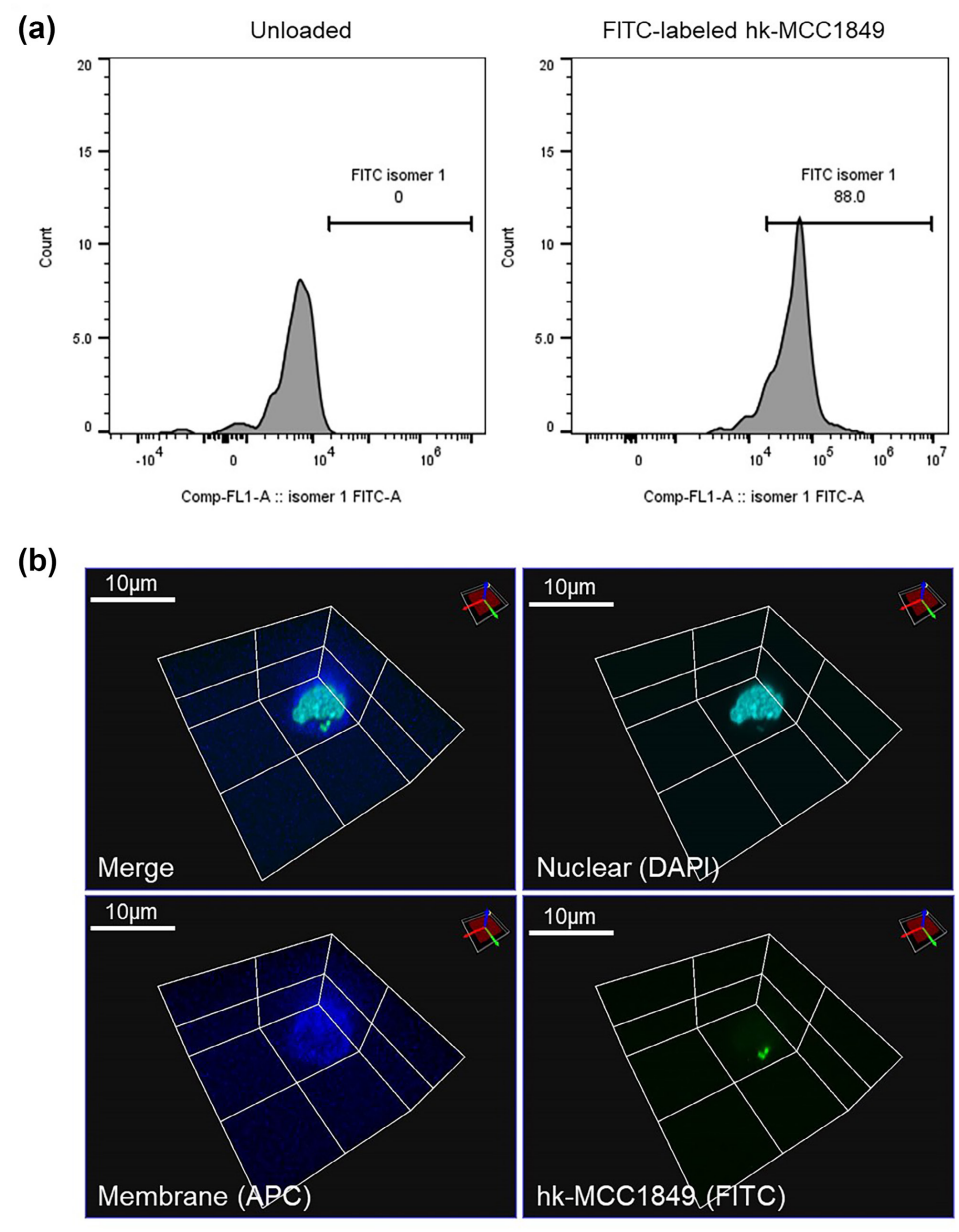
Phagocytosis of hk‐MCC1849 by pDCs. The phagocytosis of FITC‐labeled hk‐MCC1849 was detected by flow cytometry (a) and observed by laser scanning confocal fluorescence microscope (b). Scale bar: 10 μm.

Considering that pDCs account for a small proportion of normal peripheral PBMCs, to clarify whether hk‐MCC1849 directly interacts with pDCs in PBMCs, DCs from PBMCs were enriched and cocultured with FITC‐labeled hk‐MCC1849. The results showed that, compared with those in the control group, FITC fluorescence signals were increased in pDCs after 24 h of culture (Figure 3a). On the other hand, microscopic observation was also conducted to confirm the phagocytosis of hk‐MCC1849. Accumulation of FITC‐labeled hk‐MCC1849 in the cytoplasm of pDCs was observed within 24 h (Figure 3b). These results indicate that pDCs are involved in the phagocytosis of hk‐MCC1849 and suggest that the hk‐MCC1849‐pDC interaction occurred.

### 3.5 Hk‐MCC1849 directly upregulated CD86 and HLA‐DR expression in pDCs

**FIGURE 4:**
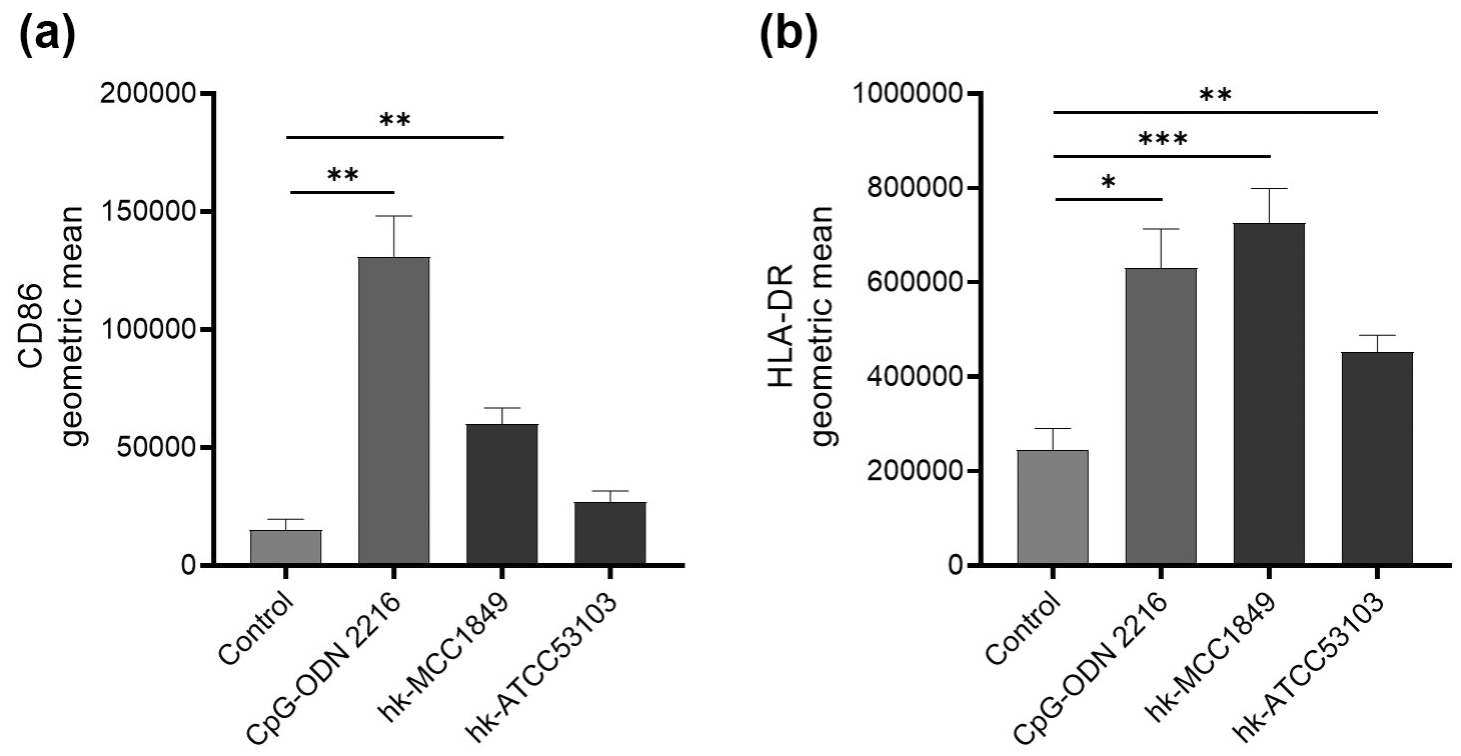
Effects of hk‐MCC1849 on surface marker expression of pDCs. CD86 (a) and HLA‐DR (b) activation in total pDCs were evaluated by FlowJo. All the data are expressed as the mean ± SE (*n* = 8). *p < .05, **p < .01, and ***p < .001 indicate a significant difference versus the control.

Furthermore, we investigated the changes in CD86 and HLA‐DR expression in pDCs using enriched DCs cocultured with hk‐MCC1849, hk‐ATCC53103, or the positive control CpG‐ODN 2216 for 24 h. The FACS results showed that significant increases in CD86 and HLA‐DR surface marker expression in pDCs were induced by both CpG‐ODN 2216 (*p* < .01 and *p* < .05) and hk‐MCC1849 (*p* < .01 and *p* < .001) . However, only significant upregulation of HLA‐DR expression (*p* < .01), but not of CD86 expression, was detected with hk‐ATCC53103 (Figure 4). These findings suggest that hk‐MCC1849 could increase the expression of CD86 and HLA‐DR in pDCs through direct interactions.

## 4 DISCUSSION

Postbiotics, which refer to the cellular components and metabolites produced by probiotics, have beneficial effects on host health. Even after the inactivation process, these key cellular components exert physiological regulatory effects, such as immunomodulation, lipid metabolism regulation, and protection against pathogens (Ishikawa et al., 2010; Noh et al., 2022; Tanaka et al., 2020), and psychological benefits, such as stress alleviation (Hirose et al., 2013; Maehata et al., 2019), in the host gastrointestinal tract. hk‐MCC1849 has been shown to strongly induce the production of IL‐12, which promotes innate immunity, after coculture with mouse spleen cells (Arai et al., 2018). In clinical trials, hk‐MCC1849 has been shown to suppress the occurrence of cold‐like symptoms in individuals who are susceptible to colds. To clarify the mechanism of the immunomodulatory effects observed in clinical studies, hk‐MCC1849 was cocultured with human PBMCs to investigate its direct effects on human immune cells in this study. The results showed that hk‐MCC1849 could be phagocytosed by pDCs; significantly elevated levels of the pDC surface markers CD86 and HLA‐DR directly; and significantly increased expression of the type I IFN genes *IFNα* and *IFNα1* after coculture. This finding suggested that hk‐MCC1849 can potentially activate human peripheral pDCs through phagocytosis.

pDCs are important cells that recognize antigens in the lumen and activate other immune cells in PPs by antigen presentation (Li et al., 2011). CD86 is an inducible costimulatory ligand of the B7 family that is constitutively expressed at a low level on monocytes (Mir, 2015). T‐cell activation requires the specific recognition of antigens presented by APCs and costimulatory signaling via CD28 engagement by CD86 on APCs (Collins et al., 2005). Costimulation of CD28 with CD80/86 is essential for the activation of CD4^+^ (Levine et al., 1996) and CD8^+^ (Seah et al., 2012) cells and for enhancing the response to viral clearance. On the other hand, the HLA molecule, which is encoded by human class II major histocompatibility complex genes, is involved in peptide antigen presentation to CD4^+^ T cells. Ahout et al. (2016) reported that infants with respiratory syncytial virus infection had reduced expression levels of HLA‐DR on monocytes in PBMC samples. The downregulation of HLA‐DR expression in peripheral blood monocytes was also observed in critically ill patients (Benlyamani et al., 2020). The activation of CD4^+^ T cells requires sufficient antigen presentation from APCs to induce a robust effector CD8^+^ T‐cell response (Bennett et al., 1997). Taken together, these previous results suggest that increased expression levels of CD86 and HLA‐DR are essential for activation of the host immune response. In the present study, Cpg‐ODN 2216 significantly induced the surface expression of CD86 and HLA‐DR on pDCs, which was consistent with previous results (Hornung et al., 2002). Additionally, significant upregulation of the surface expression of CD86 and HLA‐DR on pDCs was also detected after hk‐MCC1849 stimulation (Figures 1b,c and 4a,b). Our results indicated that hk‐MCC1849 could promote the antigen presentation of pDCs and potentially improve the host immune response.

Type I IFNs play an essential role in the activation of innate and adaptive immune responses during viral or bacterial infection by affecting B cells, T cells, and NK cells (Brinkmann et al., 1993; Fink et al., 2006; Keppler et al., 2012; Nguyen et al., 2002). In PBMCs, pDCs contribute more than 95% of type I IFN production when challenged by viral stimulation (Siegal et al., 1999). To confirm the type I IFN induction capability of pDCs, Cpg‐ODN 2216 was used as a positive control in this study. Cpg‐ODN 2216, an A‐type Cpg ODN (Vollmer et al., 2004), can efficiently trigger IFNα production by pDCs by directly interacting with TLR7/9 (Gilliet et al., 2008), which are prevalently expressed within endosomal–lysosomal compartments of pDCs and activated by viral nucleic acids (Uematsu & Akira, 2007). TLR9^−/−^ macrophages and DCs from mice were not capable of producing inflammatory cytokines in response to Cpg‐containing bacterial DNA without methylation (Hemmi et al., 2000). In this study, the phagocytosis of hk‐MCC1849 by pDCs was observed, and significant upregulation of *IFNα* and *IFNα1* expression was found. These results implied that hk‐MCC1849 might activate peripheral pDCs by interacting with TLR9. Additionally, other pathways through which pDCs respond to bacterial challenge have been reported. Raieli et al. (2019) reported that lipoproteins, major cell wall components of gram‐positive bacteria, strongly induce pDCs to produce type I IFNs through the activation of TLR2 and pro‐inflammatory cytokines through the activation of TLR1; however, contrary results showing that knockout of TLR2 does not affect type I IFN production by pDCs have been reported (Jounai et al., 2012). Considering the complexity of the bacterial structure, different extracellular molecules, such as polysaccharides and lipoteichoic acids, could affect the recognition/binding of APCs by TLR2 (Grangette et al., 2005; Miettinen et al., 2008; Sun et al., 2017) or other pathogen recognition patterns, such as C‐type lectin receptors (Sosa Cuevas et al., 2022); these molecules could also be involved in modulation of the host immune response through cooperation with other lymphocytes in PBMCs. In this study, although changes in *IFNα* expression were observed, the mechanism of interaction between pDCs and the effector component of hk‐MCC1849 is still unclear and needs to be further elucidated.

As significant changes in type I IFN gene expression were detected, we attempted to quantify the IFN concentrations in the coculture supernatants with PBMCs using an electrochemiluminescence‐based single‐PLEX assay and in the coculture supernatant with enriched DCs by the cytometric bead array method. Although a very high concentration of IFNα induced by Cpg‐ODN 2216 was detected, many data from hk‐MCC1849 and hk‐ATCC53103 were below the lower limit of detection and thus the levels were not quantified both in coculture with PBMCs and DCs. This might be a limitation of this study, and IFNα intercellular staining should be conducted in the future. Jounai et al. (2012) used mouse bone marrow‐derived Flt‐3L‐induced DCs (pDCs and myeloid DCs) and stimulated them with heat‐killed *Lactococcus lactis* subsp. *lactis* JCM5805 for 48 h, after which a high concentration of IFNα in the supernatant was detected. However, there was no significant increase in IFNα expression in pDCs purified from human PBMCs even when stimulated with the same dose of heat‐killed *L. lactis* JCM5805, likely due to the milder stimulation of human pDCs compared with murine pDCs (Sugimura et al., 2013). The addition of Cpg‐ODN 2216 strongly induced IFN production, thus indicating that the experimental system itself works well. Interestingly, a significant increase in *ISG15* gene expression was detected after hk‐MCC1849 coculture (Figure 2g). Interferon‐stimulated gene 15 (*ISG15*) is a type I IFN‐inducible protein encoded by the *ISG15* gene that plays an important role in the host antiviral response. ISG15 expression can be rapidly upregulated by both type I IFNs and several viruses (Sampson et al., 2017), and ISG15 disrupts viral replication by noncovalently binding to host proteins (Durfee et al., 2010; Tang et al., 2010; Zhao et al., 2010). This result suggested that hk‐MCC1849 potentially activates the production of type I IFNs in pDCs.

The experimental results showed that hk‐MCC1849 increased the ratio of CD86^+^HLA‐DR^+^ pDCs and upregulated the expression of the type I IFN genes *IFNα*, *IFNα1*, *IFNγ*, and *ISG15* in PBMCs. Phagocytosis of hk‐MCC1849 by pDCs was observed by coculture with enriched DCs derived from PBMCs and significant increases in the surface marker CD86 and HLA‐DR were detected in pDCs. In summary, all the results obtained from this study suggest that hk‐MCC1849 has an immunomodulatory effect on the host through the activation of peripheral pDCs.

## AUTHOR CONTRIBUTIONS

**Yiran Li:** Conceptualization (supporting); formal analysis (lead); investigation (lead); validation (lead); writing – original draft (lead); writing – review and editing (lead). **Takahiro Aoki:** Formal analysis (equal); investigation (equal); resources (equal); validation (equal). **Sadahiro Iwabuchi:** Formal analysis (equal); methodology (lead); resources (lead); validation (supporting); writing – review and editing (supporting). **Satoshi Arai:** Conceptualization (supporting); methodology (equal); writing – review and editing (equal). **Noriyuki Iwabuchi:** Conceptualization (equal); methodology (supporting); resources (equal); supervision (equal); writing – review and editing (equal). **Hideki Motobayashi:** Investigation (equal); resources (equal). **Miyuki Tanaka:** Conceptualization (lead); project administration (supporting); supervision (equal). **Shinichi Hashimoto:** Methodology (supporting); project administration (lead); resources (supporting); supervision (lead); writing – review and editing (supporting).

## CONFLICT OF INTEREST STATEMENT

We declare no conflict of interest associated with this manuscript.

## ETHICAL APPROVAL

The study was approved by the Institutional Review Board of Wakayama Medical University, Japan (Approval No. 3345).

## Data Availability

The data that support the findings of this study are available from the corresponding author upon reasonable request.
